# Polyferrocenylsilane Semicrystalline Polymer Additive for Solution-Processed *p*-Channel Organic Thin Film Transistors

**DOI:** 10.3390/polym13030402

**Published:** 2021-01-27

**Authors:** Zhengran He, Ziyang Zhang, Kyeiwaa Asare-Yeboah, Sheng Bi, Jihua Chen, Dawen Li

**Affiliations:** 1 Department of Electrical and Computer Engineering, The University of Alabama, Tuscaloosa, AL 35487, USA; zhe3@crimson.ua.edu; 2 Department of Electrical Engineering, Columbia University, New York City, NY 10027, USA; zz2284@columbia.edu; 3 Department of Electrical and Computer Engineering, Penn State Behrend, Erie, PA 16563, USA; kza56@psu.edu; 4 Key Laboratory for Precision and Non-Traditional Machining Technology of the Ministry of Education, Dalian University of Technology, Dalian 116024, China; bish@dlut.edu.cn; 5 Center for Nanophase Materials Sciences, Oak Ridge National Laboratory, Oak Ridge, TN 37831, USA

**Keywords:** small-molecule semiconductor, polyferrocenylsilane, thin film morphology, organic thin film transistors, organic electronics

## Abstract

In this study, we demonstrated for the first time that a metal-containing semicrystalline polymer was used as an additive to mediate the thin film morphology of solution-grown, small-molecule organic semiconductors. By mixing polyferrocenylsilane (PFS) with an extensively-studied organic semiconductor 6,13-bis(triisopropylsilylethynyl) pentacene (TIPS pentacene), PFS as a semicrystalline polymer independently forms nucleation and crystallization while simultaneously ameliorating diffusivity of the blend system and tuning the surface energies as a result of its partially amorphous property. We discovered that the resultant blend film exhibited a 6-fold reduction in crystal misorientation angle and a 3-fold enlargement in average grain width. Enhanced crystal orientation considerably reduces mobility variation, while minimized defects and trap centers located at grain boundaries lessen the adverse impact on the charge transport. Consequently, bottom-gate, top-contact organic thin film transistors (OTFTs) based on the TIPS pentacene/PFS mixture yielded a 40% increase in performance consistency (represented by the ratio of average mobility to the standard deviation of mobility). The PFS semicrystalline polymer-controlled crystallization can be used to regulate the thin film morphology of other high-performance organic semiconductors and shed light on applications in organic electronic devices.

## 1. Introductions

Polymeric materials as additives have rapidly attracted considerable attention for application in organic electronics [1,2,3,4,5]. When they are blended with organic semiconductors, those polymers demonstrate various merits in controlling the crystal growth, enhancing the semiconductor morphology, and improving the electrical performance of organic thin film transistors (OTFTs) [6,7]. Research efforts in the semiconductor/polymer blend systems primarily focused on various amorphous polymers such as poly(α-methylstyrene) (PαMS) [8,9], polymethyl methacrylate (PMMA) [10,11], and polystyrene (PS) [12,13]. For instance, multiple groups studied the addition of PαMS into a *p*-type small-molecular organic semiconductor 6,13-bis(triisopropylsilylethynyl) pentacene (TIPS pentacene), and observed the semiconductor vertically phase segregating with the polymer additive, which enhanced charge transport of the TIPS pentacene based OTFTs [14]. Similarly, the PS polymer was reported to blend with a high-performance organic semiconductor 2,7-dioctyl [1]benzothieno[3,2-b][1]benzothiophene (C_8_-BTBT), yielding vertical phase segregation and improved mobility [15,16,17]. In these studies, the vertical phase segregation between the organic semiconductor and amorphous polymer additive can depend on a variety of factors including solvent choice [18,19], surface energy [20,21,22,23], as well as polymer molecular weight [24,25]. Consequently, the precise control of phase segregation and the resultant compositional structure and thickness can be a technical challenge for many organic electronic device applications.

More recently, amorphous polymers have been demonstrated along with miscellaneous external alignment methods in order to fine-tune the alignment of bulk crystals and optimize the charge transport of organic semiconductors. For example, Duan et al. applied a solution shearing method to fabricate centimeter-sized C_8_-BTBT crystalline arrays with excellent alignment and an ultra-thin thickness of several molecular layers [26]. Electrical characterization of 40 C_8_-BTBT/PS based OTFTs exhibited an average saturation mobility 7-fold larger than the standard deviation, indicating improved performance consistency. Onojima et al. developed an electrostatic spray deposition (ESD) method to grow TIPS pentacene/PMMA blends on a flexible substrate [27]. The inclusion of an extra PMMA buffer layer caused a phase-segregated interface with a molecular-level flatness and enhanced charge transport properties of the organic semiconductor. Aikawa et al. reported the growth of well-oriented TIPS-pentacene/polystylene blends with vertical phase segregation by using a meniscus coating method [28]. At an optimized sweeping speed of 0.75–2.0 mm/s and an elevated temperature, reduced interfacial trap density and improved OTFT performance were both observed. Despite these advances, the complexity of experimental setup and precise parameter control of these external alignment methods may be rather challenging or even undesirable in all kinds of organic electronics applications [29].

Polymers incorporating metallic units in their chains demonstrate functions derived from both the polymers and their metallic elements, giving rise to different magnetic, redox, optical, or catalytic properties [30]. These combined merits allow metal-containing polymers to find applications in miscellaneous fields such as biosensors, electrolysis, and electro-optical devices [31]. In this study, we demonstrated for the first time the utilization of a metal-containing polymer-polyferrocenylsilane (PFS) semicrystalline polymer to control the crystal alignment, film morphology, and charge transport of solution-processed, small-molecular organic semiconductors. TIPS pentacene was employed as an example to blend with PFS because of its well-known solution processing and charge transport properties. Metal-containing polymers or metallopolymers [32] provide unique metal-involved intermolecular interactions that can result in novel electrical properties, which have not been utilized previously in mediated organic crystal growth as far as we know. Without the aid of external alignment techniques, we showed that the orientation of TIPS pentacene bulk crystals can be facilely aligned along the tilted direction of the substrate. In addition, the grain width, areal coverage, and crystal length of the TIPS pentacene were quantitatively characterized. Bottom-gate, top-contact OTFTs were fabricated by using the TIPS pentacene/PFS blends as an active layer, which demonstrates a 40% increase in device electrical performance consistency.

## 2. Experiment

TIPS pentacene and semicrystalline polymer PFS were purchased from Sigma Aldrich (St. Louis, MO, USA). Toluene was purchased from VWR (Radnor, PA, USA). All materials were used as received. Both TIPS pentacene and PFS were dissolved in toluene separately. The solute concentration was maintained at 5 mg/mL. Then the PFS solution was added into the TIPS pentacene solution at a 5% volume ratio. A simple drop casting method was used to grow the TIPS pentacene/PFS crystalline film under a “solvent rich configuration”. In this configuration, the solvent mixture was deposited onto a substrate, which was placed in a solvent-rich environment and covered with a cap. The substate was tilted at a small angle (~5°), which was intended to facilitate the crystals to align in this particular orientation.

**Figure 1:**
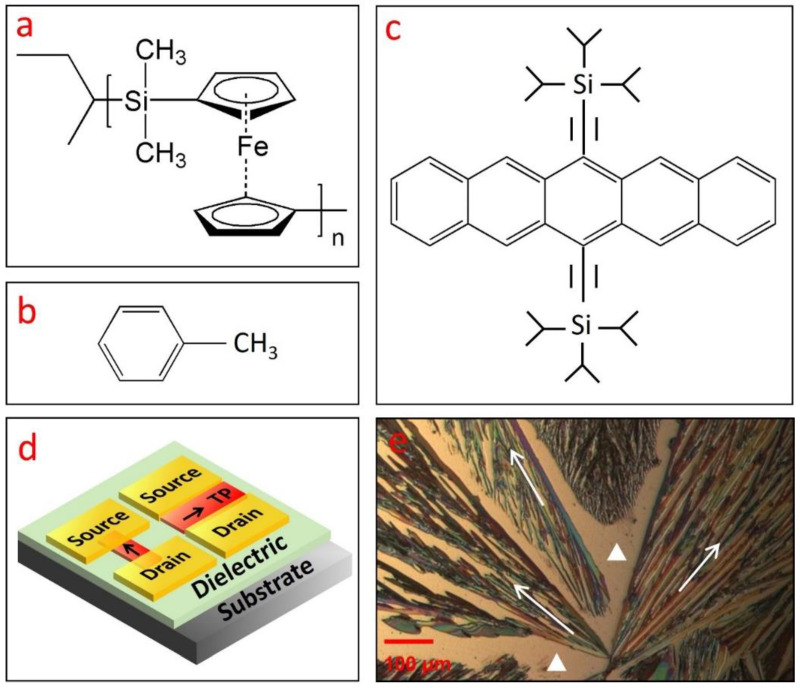
(**a**) Molecular structure of various materials used in this work, including (**a**) polyferrocenylsilane polymer (PFS), (**b**) toluene, and (**c**) 6,13-bis(triisopropylsilylethynyl) pentacene (TIPS pentacene). (**d**) A schematic drawing showing two transistors with the TIPS pentacene crystals as charge transport channel: one transistor has a crystal orientation parallel to the direction from the Au source to drain electrodes, while the other has a crystal orientation perpendicular to this direction. (**e**) Polarized microscopic picture of TIPS pentacene film without the PFS additive, which was grown via simple drop casting in toluene solution. The pristine TIPS pentacene crystals exhibited dendritic patterns of crystal growth and significant crystal misorientation. The crystal orientation or charge transport direction of TIPS pentacene is shown by the arrows in (**d**,**e**). The bare substrate in (**e**) is marked by the two white triangles.

The fabrication of bottom-gate, top-contact OTFTs was based on a silicon substrate with a thermally grown silicon dioxide layer (250 nm) as dielectric. Gold (Au) was deposited via thermal evaporation as source and drain electrodes with a thickness of 50 nm, as shown in the device configuration of Figure 1d. The thermal deposition of Au had an evaporation rate of 1 Å/s under a pressure of 10^−7^ Torr. The channel dimensions were defined by the shadow mask, which has a width of 2000 μm and different lengths of 25, 50, 75, and 100 μm. The optical images of TIPS pentacene/PFS blend crystals were taken by using a polarized optical microscope. The electrical performance of TIPS pentacene/PFS based OTFTs was characterized by using a Keithley 4200 semiconductor (Keithley Company, Cleveland, OH, USA) parameter analyzer. Measurement of each device was repeated five times in order to ensure the consistency of results. All characterization took place at room temperature in an ambient environment.

## 3. Results and Discussion

The molecular structures of the PFS polymer and toluene are shown in Figure 1a,b, respectively. The incorporation of iron substitution in the PFS polymer bestows unique properties, including redox activity, semicrystallinity, and photoconductivity, which are not accessible by traditional organic polymers [33,34,35,36]. In addition, the precise control of chain length in the metallopolymers also greatly expands its potential applications. Symmetrical substitution often results in PFS polymer with a semicrystalline nature, whereas asymmetrical substitution generally leads to PFS polymer with an amorphous nature [37]. As compared to purely organic polymers, the presence of silicon and iron in the polymer main chain enhances the stability of the PFS polymer, which allows it to be potentially applied in plasma-assisted reactive ion etching as barrier material [38]. The high refractive index and nonconductivity of the PFS polymer makes it promising for applications in light-emitting diodes, antireflection coating [39], as well as satellite coating to prevent arc discharge [40].

The molecular structure of the small-molecular organic semiconductor TIPS pentacene is shown in Figure 1c. As a derivative of pentacene, TIPS pentacene exhibits enhanced charge transport due to its π–π stacking structure. The addition of the bulky side groups interrupts the herringbone packing patterns and improves the solubility of TIPS pentacene in organic solvents [41,42,43,44]. TIPS pentacene was employed as a benchmark material to mix with PFS in this work due to its enhanced solubility and charge transport properties as mentioned above. When drop casted in solution, TIPS pentacene exhibits a dendritic growth pattern, as shown in the polarized optical image of Figure 1e. We use the arrows to represent the long axis of the TIPS pentacene, which is also parallel to the charge transport direction in the crystals [45]. The white triangles refer to the substrate without TIPS pentacene coverage. It can be clearly seen that the crystal orientations are essentially random, lacking order and alignment over a long range. With such misoriented crystals as the charge transport channel, the mobility measured from the OTFTs can undergo significant variations [46].

We employ the schematic plot in Figure 1d as an illustration to explain the interplay among crystal orientation and mobility variation of the OTFTs. Figure 1d shows the device configuration of two bottom-gate, top-contact OTFTs that share the same substrate. TIPS pentacene (abbreviated as “TP”) crystals with different crystal orientations are incorporated in the charge transport channel. In particular, one transistor device has crystal orientation (presented by the arrows) parallel to the direction from source to drain contact electrodes, whereas the other device has crystal orientation perpendicular to this direction. A previous study employed an “anisotropic ratio” to examine the effect of crystal misorientation on the extracted mobility of solution-processed TIPS pentacene thin films [47]. By systematically changing the angle between the crystal long axis [210] and source-to-drain direction, an anisotropic ratio of up to 10 was demonstrated, indicating that the extracted mobility can exhibit a significant difference of up to 10-fold. The OTFTs as depicted in Figure 1d can thereby undergo dramatic performance inconsistency, which restricts the potential applications in organic electronics.

**Figure 2:**
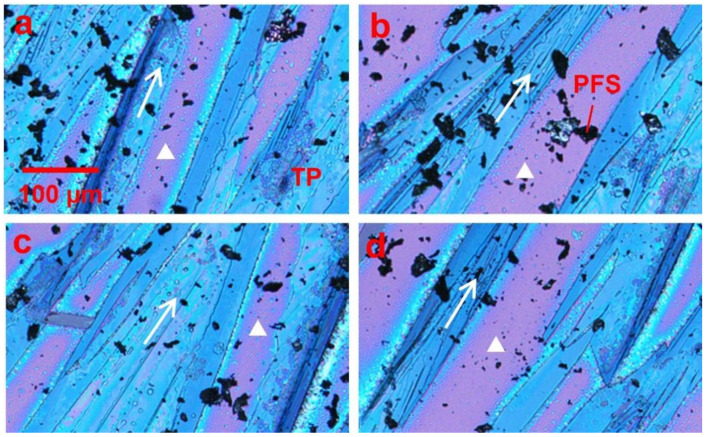
Polarized optical microscopic images showing the effect of the loading of the PFS polymer on the thin film morphology of TIPS pentacene crystals. All images in (**a**–**d**) were taken from different sections of the substrate, which showed that the PFS polymer minimized the dendritic pattern of TIPS pentacene crystal growth. Instead, the TIPS pentacene/PFS crystals were aligned more uniformly with larger grain width and coverage. The arrows in all four images indicate the crystal orientation. The white triangles represent the surface without the TIPS pentacene/PFS film. All images share the same scale bar as (**a**).

In order to regulate the dendritic growth pattern and mobility variation of TIPS pentacene, we mixed it with the PFS semicrystalline polymer as an additive. The resultant thin film morphologies of the TIPS pentacene/PFS blend crystals are presented in Figure 2. The polarized optical images in Figure 2a–d were taken from different sections of the same substrate. The arrows represent the long axis [210] or the charge transport direction of TIPS pentacene crystals, while the white triangles indicate the bare substrate without TIPS pentacene or PFS coverage. As observed under a polarized light microscope, TIPS pentacene domains exhibit color variants induced by crystal orientations with a longitudinal dimension extending to hundreds of microns [48]. On the other hand, as a semicrystalline polymer, PFS forms crystals as a result of its own nucleation and crystallization, giving rise to the formation of the relatively dark crystals shown in the optical images. The optical micrographs only show the PFS domains that are large enough for optical images. Scanning electron microscopy (SEM) and transmission electron microscopy (TEM) on nanoscale polymer/organic semiconductor interfaces have many challenges including electron beam induced sample damage and lack of contrast, which were not pursued in this work. This result clearly indicates that the crystal orientations of TIPS pentacene are strictly aligned towards a more uniform direction (the same as the tilted direction of the substrate) due to adding the PFS semicrystalline polymer. 

**Figure 3:**
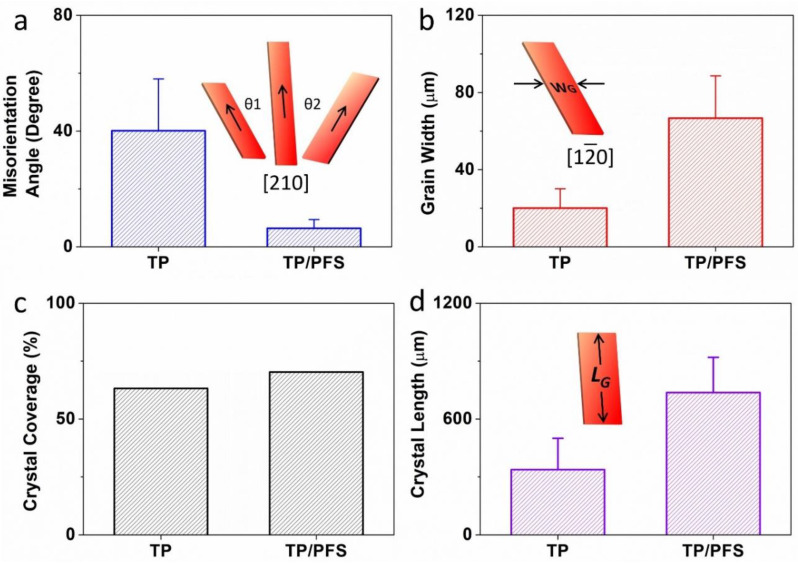
The addition of the PFS polymer had a significant impact on the misorientation angle (θ), grain width (abbreviated as W_G_), crystal coverage, and crystal length (abbreviated as L_G_) of the TIPS pentacene crystals. (**a**) θ_1_ and θ_2_ are the misorientation angles, which were measured based on the angle between the charge transport direction (equivalent to the long axis [210]) of one crystal and that of a baseline crystal. (**b**) W_G_ is the grain width, equivalent to the crystal width along the [1$\bar{2}$0] short axis of TIPS pentacene. The average and standard deviation of both misorientation angle and grain width are based on 18 measurements. (**c**) The crystal coverage refers to the ratio of the substrate that was covered by the TIPS pentacene crystals. (**d**) The crystal length was measured along the same long axis [210] as indicated in (**a**). The average and standard deviation of crystal length were based on 10 measurements.

To more accurately characterize how the addition of the PFS polymer enhances the thin film morphology of TIPS pentacene, we quantitatively calculated the following important parameters of the blend film: the misorientation angle, grain width, crystal coverage, and crystal length. As plotted in Figure 3a, the misorientation angle θ_1_ and θ_2_ is designated as the angle between the long axis [210] (represented by the arrows) of the TIPS pentacene crystals. The calculation of misorientation angles was based on 18 measurements for both pristine TIPS pentacene film and TIPS pentacene/PFS blend film. Since the crystal long axis dictates the direction of charge transport, the misorientation angles can adequately characterize the uniformity of charge carrier mobility as a result of adding PFS as a semicrystalline polymer additive. The pristine TIPS pentacene film exhibits a large misorientation angle of 40.1° ± 17.9°, as depicted in Figure 3a. In comparison, the TIPS pentacene/PFS blend film demonstrates a greatly reduced misorientation angle of 6.4° ± 3°. The result clearly indicates that blending PFS as an additive yielded a 6-fold reduction in the crystal misorientation, which confirms the observation of enhanced crystal alignment in a long-range order as shown in the polarized optical images. 

We continue to characterize the change of the TIPS pentacene crystal grain width as a result of adding PFS as a semicrystalline polymer. As illustrated in the inset of Figure 3b, the grain width refers to the crystal width measured along the crystal short axis [1$\bar{2}$0]. Based on 18 measurements, the pristine TIPS pentacene film exhibits an average grain width of 20.1 ± 10 µm, whereas the TIPS pentacene/PFS blend film shows an enlarged grain width of 66.7 ± 21.9 µm. Evidently, the addition of the PFS semicrystalline polymer results in a more than 3-fold increase of the grain width of TIPS pentacene crystals. It is well known that the defects and charge trap centers can be mostly found at the grain boundaries of the crystals [49,50,51,52], and thus, less populated grain boundaries as a result of enlarged grain width can essentially benefit the electrical performance of the small-molecular semiconductor based transistor.

In addition, the crystal coverage was calculated based on the percentage of the substrate that was covered with the semiconductor crystalline film. As plotted in Figure 3c, the pristine TIPS pentacene had a coverage of 63.2%, whereas the TIPS pentacene/PFS blend film exhibits a higher coverage of 70.3%. Note that the crystal coverage was only based on the crystalline TIPS pentacene, without including the dark PFS crystals. The crystal length was measured along the long axis [210] of the TIPS pentacene crystals. As shown in Figure 3d, the pristine TIPS pentacene showed an average crystal length of 337.4 ± 162.6 µm, while the addition of the PFS polymeric additive enlarged the crystal length to 737.2 ± 182.7 µm, which is more than a 2-fold increase as compared to the pristine film. Based on the morphological characterization as stated above, it can thus be inferred that the PFS polymeric additive effectively attributed to the formation of long-stretching, strictly-aligned TIPS pentacene crystals with improved film uniformity, larger grain width, and higher substrate coverage.

**Figure 4:**
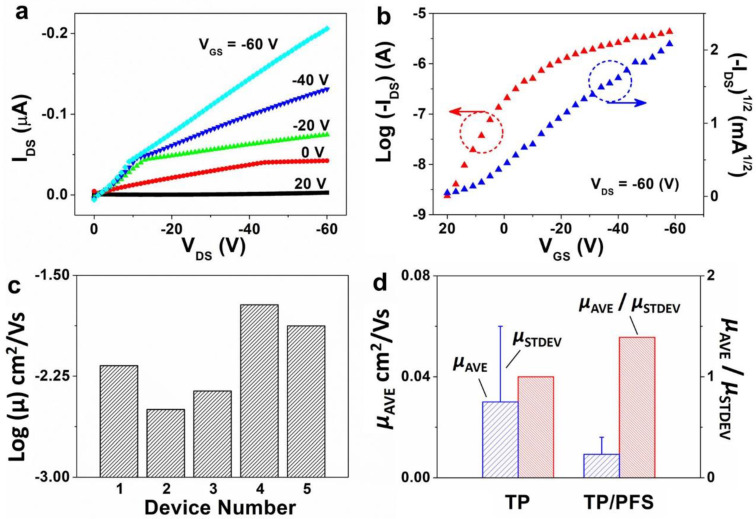
Electrical characterization results of top-contact, bottom-gate OTFTs fabricated based on the TIPS pentacene/PFS mixture. (**a**,**b**) show the typical output and transfer curves measured from the devices. The measurement of all OTFT devices was repeated five times ensure the consistency of mobility extraction. (**c**) Log-scale plot of the extracted field-effect mobility from different TIPS pentacene/PFS based OTFT devices located on the same substrate, showing reduced mobility variations. (**d**) A plot showing the average mobility of OTFTs based on pristine TIPS pentacene and TIPS pentacene/PFS blends. The ratio of average mobility to the standard deviation of mobility is used as a metric to evaluate the performance consistency of OTFT devices.

We fabricated TIPS pentacene/PFS OTFTs based on a top-contact, bottom-gate configuration. As compared to bottom-contact counterparts, the top-contact devices have the merit of simplified fabrication, eliminating the complexity and high cost of contact electrode patterning by using standard photolithography. The electrical characterization of the TIPS pentacene/PFS devices, including both output and transfer curves, is presented in Figure 4a,b. The output curve was measured by sweeping the source-to-drain voltage *V_DS_* from 0 to −60 V, while changing the gate voltage *V_GS_* from 20 to −60 V with a step of -20 V. The transfer curve was measured by sweeping the *V_GS_* from 20 to −60 V, while maintaining the *V_DS_* at −60 V. The output curve did not exhibit saturation behavior as the $V_{DS}$ increased to −60 V, which can be presumably attributed to the incorporation of iron in the PFS polymer chains. From the square root plot (${\left(-I_{DS}\right)}^{1/2}\sim V_{GS}$) in the transfer curve, we calculated the hole mobility of TIPS pentacene/PFS blends based on the square-law model of Equation (1):

(1) $$I_{DS}=\mu C_{i}\frac{W}{2L}{\left(V_{GS}-V_{T}\right)}^{2}$$

In the equation stated above, *μ* represents the mobility to be extracted, *I_DS_* represents the saturation drain current, *V_GS_* is the applied gate-source voltage, *V_T_* is the threshold voltage, *C_i_* is the gate dielectric capacitance (13.8 nF/cm^2^), and *W* and *L* are the shadow mask width and length.

The field-effect mobility of the TIPS pentacene/PFS blend based OTFTs is plotted in a log scale in Figure 4c. The mobility of five different devices of the same substrate was extracted as 3.2 × 10^−3^, 4.4 × 10^−3^, 6.7 × 10^−3^, 1.3 × 10^−2^, and 1.9 × 10^−2^ cm^2^/Vs, which indicates that the mobility from all OTFTs only varies within one order of magnitude. In contrast, the mobility of pristine TIPS pentacene OTFTs without adding the PFS polymeric additive dramatically ranged between 9.8 × 10^−5^ and 8.4 × 10^−2^ cm^2^/Vs [53], showing mobility variations of 3 orders of magnitude. The average mobility of TIPS pentacene OTFTs without and with adding the PFS semicrystalline polymer was 3 × 10^−2^ ± 3 × 10^−2^ [53] and 9.33 × 10^−3^ ± 6.7 × 10^−3^ cm^2^/Vs, respectively, as shown in the plot of Figure 4d. Furthermore, we employed the ratio of average mobility (*µ_AVE_*) to standard deviation (*µ_STDEV_*) of mobility as an effective measure to quantitatively evaluate the impact of mobility variation on the performance consistency of OTFTs. The *µ_AVE_*/*µ_STDEV_* ratio was calculated to be 1 and 1.4 based on pristine TIPS pentacene film and TIPS pentacene/PFS blend film as the active layer of OTFT devices, respectively. It can therefore be inferred that the mixing of PFS semicrystalline polymer additive attributed to a 40% enhancement of performance consistency of the TIPS pentacene OTFTs.

The mobility with the addition of PFS polymer was slightly lower than but still comparable to the values for the pristine TIPS pentacene. It is important to emphasize that the extracted mobility can depend on a few important factors. First, we intentionally used a large channel dimension in this work (the channel width was 2000 µm, whereas the channel length was up to 100 µm), whereas the pristine TIPS pentacene based OTFTs had a much shorter channel width of 1000 µm. Such a large channel dimension in this work may inevitably incorporate more crystalline defects and trap centers in the charge transport channel, which reduces the mobility extracted from the TIPS pentacene/PFS blend film. Second, the channel width and length used in Equation (1) for extracting mobility were directly based on the dimension of the shadow mask, which can result in lower mobility when the charge transport channel does not have a full coverage. This flatly contradicts many previous studies that used the actual channel dimensions only with crystal coverage for mobility calculation [54,55,56]. Third, no surfactant treatment was applied to the OTFT device in this work. For example, a pentafluorobenzene thiol (PFBT) surfactant treatment is typically employed on the contact electrodes to tune the work functions and to facilitate charge injection [57]. The bottom-gate, top-contact configuration used in this work involves the deposition of the organic semiconductor layer prior to the evaporation of contact electrodes, which does not allow the utilization of PFBT treatment without damaging the active layer. Hexamethyldisilazane (HMDS) treatment [58] can also be applied to the substrate to passivate the hydrophilic silanol groups, which would otherwise serve as charge trap centers and undermine the electrical performance of the TIPS pentacene/PFS based OTFTs. Therefore, the mobility reported in this work can be significantly enhanced by utilizing and optimizing these factors as mentioned above.

**Figure 5:**
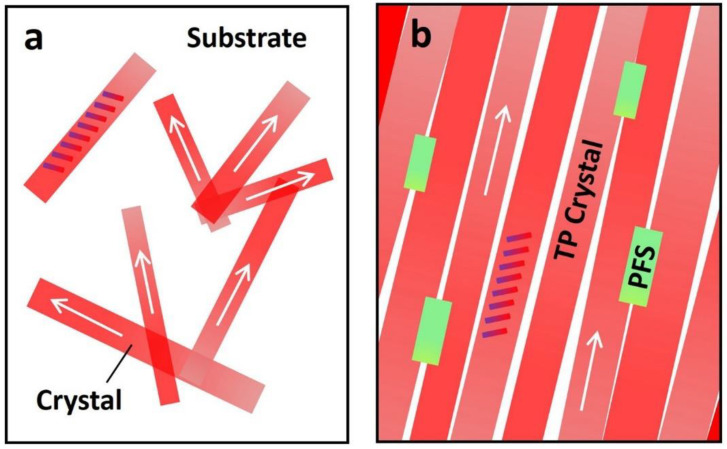
Schematic plots depict (**a**) the dendritic pattern of TIPS pentacene crystal growth, showing random orientation, small grain width, and low substrate coverage; (**b**) TIPS pentacene/PFS film showing elimination of the TIPS pentacene dendritic growth pattern, remarkable crystal alignment, enlarged grain width, and enhanced coverage of the mixed film on the substrate. The semicrystalline PFS can nucleate and crystallize independently of the crystallization events of TIPS pentacene. The tilted rods indicate how the TIPS pentacene backbones are oriented. The arrows indicate the long axis [210] orientation of TIPS pentacene.

Finally, we use the plots in Figure 5 to illustrate the interplay among polymer additive, thin film morphology, and charge transport. The arrows in this figure represent the long axis [210] or charge transport direction of TIPS pentacene. The titling rods indicate the backbone orientation of TIPS pentacene. The pristine TIPS pentacene growth can be predominantly characterized by a dendritic pattern, resulting in crystals with random directions and low substrate coverage, as shown in Figure 5a. In contrast, the PFS semicrystalline polymer additive effectively enhances the TIPS pentacene thin film morphology by reducing the crystal misorientation and enlarging the grain width. As a result, the TIPS pentacene/PFS blend film exhibits straight alignment of crystals that extend in a long-range order, as shown in Figure 5b. The mechanism of the PFS polymer additive modulating the thin film morphology of TIPS pentacene can be explained by the following aspects. First, similar to previously reported amorphous polymers such as PαMS, PS, and PMMA, the partially amorphous property of PFS can exert a strong effect on ameliorating diffusivity of the blend system and tuning the surface energies. Second, the hydrophobic nature of the PFS polymer core [59] is likely to induce intermolecular interactions with the hydrophobic alkyl side groups of TIPS pentacene, which promotes uniform deposition of nucleation seeds and enhances homogeneous crystallization of the organic semiconductor [43]. The ferrocene of PFS core can also have steric and electromagnetic effects that are not available in other polymer additives in guiding the TIPS pentacene crystallization. Third, as a semicrystalline polymer, PFS independently forms nucleation and crystallization. These effects synergistically attribute to the enhanced crystal alignment, improved film coverage, and enlarged crystal size of TIPS pentacene. Unlike conjugated polymers, which promote π–π interactions and cocrystallization with the organic semiconductor [60,61,62], the PFS eliminates such complicated processes, which can be otherwise undesirable for many facile processes. Furthermore, in Table 1, we summarize miscellaneous properties of PFS [63,64,65] and compare them with those of PS. These important properties include the density, glass transition temperature, crystallization behavior, Mark–Houwink parameter and potential applications. Therefore, the employment of the PFS additive allows a synergistic combination of the respective advantages from both semiconducting polymers and amorphous polymers, which can give rise to broader applications in various fields.

## 4. Conclusions

In summary, we report the employment of a semicrystalline polymer PFS to alleviate random orientation, improve crystal alignment, and enlarge grain width of the small-molecular organic semiconductor TIPS pentacene. Blending PFS as an additive yielded a 6-fold reduction in the crystal misorientation angle to 6.4° ± 3° and was simultaneously attributed to a 3-fold enhancement of the grain width to 66.7 ± 21.9 µm. Consequently, TIPS pentacene crystals were strictly aligned towards a uniform orientation and stretched in a long-range order across the substrate. Such morphological enhancement was attributed to the amelioration of the blend system diffusivity and the modification of surface energies due to the partially amorphous property of PFS. Bottom-gate, top-contact TIPS pentacene/PFS based OTFTs demonstrated a mobility of up to 1.9 × 10^−2^ cm^2^/Vs and a remarkable 40% enhancement in performance consistency (denoted by the ratio of average mobility to the standard deviation of mobility). We believe PFS would also exert similar effects on modulating the thin film morphology and charge transport of other small-molecular organic semiconductors. Future efforts can be dedicated to studying the possible phase segregation between TIPS pentacene and PFS, which will shed light on their application in organic electronics devices on flexible substrates. 

## Figures and Tables

**Table 1:** Miscellaneous properties of PFS and PS [63,64,65].

Property	PFS	PS
Density	1.3 g/cm^3^	1.05 g/cm^3^
Glass Transition Temperature	33 °C	100 °C
Crystallization Behavior	Spherulitic crystal growth; single crystal: trans-planar zigzag with parallel packing; films and fibers: monoclinic crystalline polymer phase along with a hexagonal or tetragonal mesophase	Amorphous film
Mark–Houwink Parameter	0.62 (in THF), random coils of PFS much denser in THF than those of PS	0.72 (in THF)
Applications	Magnetic nanostructures	Pharmaceutical, medical, horticulture, appliance packaging, etc.

## Data Availability

Not applicable.
